# Epidemiological aspects of HIV infection in the Republic of Moldova

**DOI:** 10.1186/1471-2334-14-S4-P8

**Published:** 2014-05-29

**Authors:** Iurie Osoianu, Ala Halacu

**Affiliations:** 1 National Center of Public Health, Republic of Moldova

## 

HIV infection in the Republic of Moldova continues to represent a major problem of public health, which is kept under constant surveillance and monitoring. On January 1, 2014 the cumulative number of persons identified with HIV was 8,588 people, representing a prevalence of 166.74 cases per population of 100,000. In 2013, HIV incidence was 17.18 cases per 100,000 population. Cumulative AIDS was diagnosed in 2,464 persons (28.7% of people diagnosed HIV positive). 1,752 people died at the onset of the epidemic (20.5% of all people diagnosed with HIV).

The HIV epidemic evolution in the country is characterized by three periods with some features:

- 1987-1995 registering sporadic cases in some areas, mainly among foreign students - sexually transmitted ways;

- 1996-2001 expanding geographic areas and spreading predominantly among injecting drug users (IDU), the route of transmission through injecting drug use;

- 2002-present spreading in all administrative territories, including in rural areas, increasing the number of persons infected sexually, increasing the percentage of impaired women.

The incidence of HIV in Moldova has increased significantly since 2003. So far, the epidemic has affected most intravenous drug users (IDU), commercial sex workers (CSW), men who have sex with men (MSM) and their partners, thus being classified as epidemic outbreak concentrated in population groups with high risk of infection. The country is assured access to preventive services to all population groups, especially IDU, CSW and MSM. It is also ensured access to treatment, care and support for HIV infected persons and AIDS patients.

